# Adjuvant Therapies in Diabetic Retinopathy as an Early Approach to Delay Its Progression: The Importance of Oxidative Stress and Inflammation

**DOI:** 10.1155/2020/3096470

**Published:** 2020-03-11

**Authors:** Ricardo Raúl Robles-Rivera, José Alberto Castellanos-González, Cecilia Olvera-Montaño, Raúl Alonso Flores-Martin, Ana Karen López-Contreras, Diana Esperanza Arevalo-Simental, Ernesto Germán Cardona-Muñoz, Luis Miguel Roman-Pintos, Adolfo Daniel Rodríguez-Carrizalez

**Affiliations:** ^1^Institute of Clinical and Experimental Therapeutics, Department of Physiology, Health Sciences University Center, University of Guadalajara, Guadalajara, Jalisco, Mexico; ^2^Department of Ophthalmology, Specialties Hospital of the National Occidental Medical Center, Mexican Institute of Social Security, Guadalajara, Jalisco, Mexico; ^3^Department of Ophthalmology, Regional General Hospital 110, Mexican Institute of Social Security, Guadalajara, Jalisco, Mexico; ^4^Department of Ophthalmology, Hospital Civil de Guadalajara “Fray Antonio Alcalde”, Guadalajara, Jalisco, Mexico; ^5^Department of Physiology, University Center of Tonala, University of Guadalajara, Tonala, Jalisco, Mexico

## Abstract

Diabetes mellitus (DM) is a progressive disease induced by a sustained state of chronic hyperglycemia that can lead to several complications targeting highly metabolic cells. Diabetic retinopathy (DR) is a multifactorial microvascular complication of DM, with high prevalence, which can ultimately lead to visual impairment. The genesis of DR involves a complex variety of pathways such as oxidative stress, inflammation, apoptosis, neurodegeneration, angiogenesis, lipid peroxidation, and endoplasmic reticulum (ER) stress, each possessing potential therapeutic biomarkers. A specific treatment has yet to be developed for early stages of DR since no management is given other than glycemic control until the proliferative stage develops, offering a poor visual prognosis to the patient. In this narrative review article, we evaluate different dietary regimens, such as the Mediterranean diet, Dietary Pattern to Stop Hypertension (DASH) and their functional foods, and low-calorie diets (LCDs). Nutraceuticals have also been assessed in DR on account of their antioxidant, anti-inflammatory, and antiangiogenic properties, which may have an important impact on the physiopathology of DR. These nutraceuticals have shown to lower reactive oxygen species (ROS), important inflammatory factors, cytokines, and endothelial damage biomarkers either as monotherapies or combined therapies or concomitantly with established diabetes management or nonconventional adjuvant drugs like topical nonsteroidal anti-inflammatory drugs (NSAIDs).

## 1. Introduction

Diabetic retinopathy (DR) is the number one cause of blindness in people between 26 and 75 years of age, and it is estimated that 191 million people will be diagnosed with DR by 2030 [1]. DR is an often-overlooked complication of diabetes mellitus (DM); however, diabetes-related blindness may cost up to $500 million annually in the US. This may be because of the limited therapeutic options that exist to lessen its progression in early stages [2], which emphasizes even more the importance of an early, effective approach that would offer a much better prognosis to the patient. Other nonconventional therapies have been reviewed widely as novel treatment; however, none has shown any clinical significance to change the expectant approach from specialized ophthalmologists [3]. Until now, ophthalmologists prefer to optimize glycemic control and other comorbidities with a one-year recall until there is evidence of proliferative diabetic retinopathy (PDR).

In this narrative review, we aim to approach different diets and their effects on DR and different nutraceuticals that have shown antioxidant and anti-inflammatory properties proven to have different outcomes in the genesis and development of DR. To achieve this, we searched the published literature through online databases such as Embase, PubMed, Scopus, Web of Science, Google Scholar, and Science Direct, using the following search terms: “diabetic retinopathy,” “adjuvant therapy,” “inflammatory,” “inflammation,” “oxidative stress,” “antioxidant therapy,” “diet,” “dietary,” “supplementation,” “nutraceuticals,” “combined therapy,” “dash,” “Mediterranean,” “low-calorie,” “NSAID,” “bromfenac,” “nepafenac,” “topical,” “fenofibrates,” “brimonidine,” “captopril,” and “angiotensin receptor”; we included English language articles that showed relevance either in clinical or in preclinical stages in DR, with the oldest article being from 1992 up to November 2019. We took into consideration articles that contribute to the discussion of adjuvant therapies to stabilize or to delay the progression of DR. We are considering an adjuvant therapy as a nontraditional or nonstandardized “add on” treatment [4] that acts in the physiopathological mechanisms of DR, taking into consideration that these do not replace the established DM management.

## 2. Physiopathology

DR is a complex disease that comes from the alteration of various pathways affecting the retina. The retina is susceptible to damage from high-glucose concentrations by being a highly metabolically active tissue, especially after 6 years of diagnosis [5]. Chronic hyperglycemia is the main risk factor involving DR, and it has been shown to induce vascular endothelial dysfunction in the retina [6] by damaging pericytes. If injury by high glucose persists, other pathways besides glycolysis (such as polyol, hexosamine, and advanced glycation) will be activated, the latter known to induce apoptosis and degeneration of pericytes, and thus, the retina will be damaged over time [7]. These changes can be clinically observed with ophthalmoscope-like microaneurysms, intraretinal microvascular abnormalities, and neovascularization leading to formation of exudates and hemorrhages [8].

**Figure 1:**
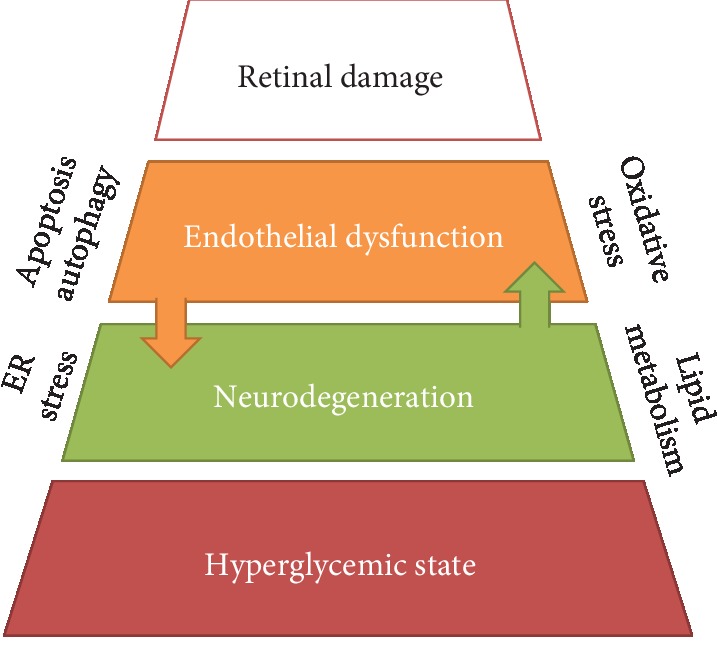
Diabetic retinopathy physiopathology pyramid. This is a representation of DR's pathophysiology on how every step leads to the next upper one. Hyperglycemic state is the first step, followed by neurodegeneration and endothelial dysfunction, underlying oxidative stress, endoplasmic reticulum (ER) stress, lipid metabolism abnormalities, apoptosis, and autophagy as a complex interconnected pathophysiology leading to retinal damage in DR.

According to previous research, independent metabolic pathways are connected between hyperglycemia and DR [9] (see Figure 1). Oxidative stress (OS) is associated with inflammation [10]; mitochondrial dysfunction, apoptosis, and DNA repair system dysfunction [11]; and neurodegeneration due to the formation and augmented concentration of reactive oxygen species (ROS) [1], with the latter inducing inflammation and mitochondrial dysfunction ultimately leading to cell death [12]. OS has been one of the most studied therapeutic approaches besides endothelial dysfunction.

**Figure 2:**
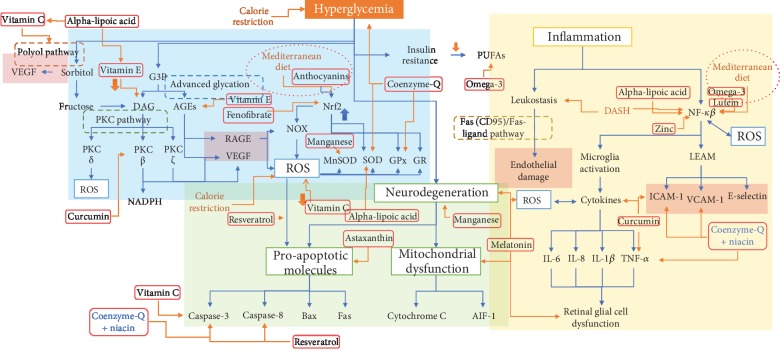
DR's physiopathology and biomarkers for each intervention. Hyperglycemia induces a variety of biochemical responses derived in angiogenesis (as shown by VEGF and endothelial damage), apoptosis, and retinal dysfunction; here, we describe where each intervention acts in these complex pathways by inhibiting the shown biomarker. Abbreviations: VEGF: vascular endothelial growth factor; G3P: glyceraldehyde 3-phosphate; DAG: diacylglycerol; AGEs: advanced glycation end products; RAGE: receptor for advanced glycation end products; PKC: protein kinase C; NOX: NADPH (nicotinamide adenine dinucleotide phosphate) oxidase; ROS: reactive oxygen species; SOD: superoxide dismutase; MnSOD: manganese superoxide dismutase; GPx: glutathione peroxidase; GR: glutathione reductase; ICAM-1: intercellular adhesion molecule-1; VCAM-1: vascular cell adhesion molecule-1; MCP-1: monocyte chemoattractant protein-1; TNF-*α*: tumor necrosis factor-alpha; IL-6: interleukin-6; IL-8: interleukin-8; IL-1*β*: interleukin-1*β*; PUFAs: polyunsaturated fatty acids; AIF-1: apoptosis-inducing factor-1; Bax: Bcl-2-associated X protein; LEAM: leukocyte-endothelium adhesion molecules; DASH: Dietary Pattern to Stop Hypertension.

Another important and widely studied pathway involved in DR is inflammation, where many cytokines: interleukin-1*β* (IL-1*β*), interleukin-6 (IL-6), interleukin-8 (IL-8), tumor necrosis factor-*α* (TNF-*α*), and monocyte chemoattractant protein-11 (MCP-11) as seen in Figure 2, have been reported in ocular tissues in preclinical and clinical studies. Inflammation correlates with an increased concentration in PDR over early stages [13]. Lipid metabolism is a biomechanism that plays an important role in the genesis of DR; for example, large omega-3 polyunsaturated fatty acids (PUFAs) play a regulating role on vascular function and angiogenesis as shown by Eid and colleagues [14]. Other mechanisms are also described, such as endoplasmic reticulum (ER) stress where the proteostasis mediated by chronic conditions such as DM activates signaling pathways as protein kinase R/PKR-like ER kinase from unfolded protein response, and autophagy is overwhelmed, leading to cell death [15].

## 3. Dietotherapy in Diabetic Retinopathy

Nutrition plays an important role in controlling the diabetes progression and its complications [16]. Optimizing glycemic and lipid control is one of the first-line interventions in diabetes, which also reduces the progression of DR [17]. However, here, we will focus on antioxidant-rich specific diets and supplements as an adjuvant therapy to the usual diabetes management.

Specific recommendations on diet, its components, or different nutraceuticals exerting their effects on type II diabetes mellitus (T2DM) have been reviewed. As an example, there are very low-calorie diets (VLCD) [18–20], which have shown to be effective on weight and glycemic control on patients with T2DM.

Other components of an everyday diet worth mentioning that have already been discussed regarding DR are PUFAs, which are associated with reduced prevalence and progression of DR [21]. A moderate consumption of alcohol [22], especially white and fortified wines [23], has also been studied with inconclusive results on the effects in DR according to different cohorts [24, 25]; and progressive studies [26] show no association between their consumption and the onset or progression of DR. Furthermore, high sodium intake, an important aspect of the Dietary Pattern to Stop Hypertension (DASH) diet, may be related with progression of DR, showing correlation in patients with type I diabetes mellitus (T1DM) [27], but not showing an association in other progressive studies [28] nor in patients with T2DM [29]. Carbohydrate intake independently has proven not to be associated with DR [30], which brings attention to the importance of understanding the pathophysiological mechanisms involved in the genesis and progression of DR, suggesting that its treatment requires a different approach other than diabetes management alone.

Mediterranean diet is a recognized overall healthy diet, and it has been shown to have a protective effect against DR [31, 32]. It contains a high amount of fish and extra virgin olive oil rich in omega-3 PUFAs [33] and mixed nuts rich on polyphenols that may reduce the risk of developing diabetes [34] and benefits insulin resistance [35] (see Table 1). This diet is rich in anti-inflammatory and antiangiogenic factors such as the nuclear factor erythroid 2-related factor 2 (Nrf2) which also increases antioxidant enzyme activity [36] (as seen in Figure 2). Augmented intake of vitamin-rich food as fruits and vegetables, as well as supplements, has been related to a risk reduction of chronic diseases [37] and DR itself [38]. This high fruit and vegetable consumption has shown hypoglycemic effects due to their bioactive compounds such as flavonoids, alkaloids, and anthocyanins being present in wild blueberry, bilberry, cranberry, elderberry, raspberry seeds, and strawberry which have shown to have powerful antioxidant activity [39]. Other micronutrients such as vitamins C and E have not shown an association between DR risk and intake of these vitamins [40] in contrast to a prospective study on fruit consumption and DM and its vascular complications [41]. Mediterranean diet through its dietary components acts in dyslipidemia, inflammation, oxidative stress, and hyperglycemia through some of the nutraceuticals discussed in this article which we focus as potential monotherapy or combined therapy agents [42].

Coffee and caffeine have been studied in DR, and conclusiveness has not yet been achieved. A cohort study conducted by Lee et al. in 2016 concludes that drinking coffee without milk or sugar at least three times daily has a preventive effect on diabetes [43]. Such influence is associated with chlorogenic acid on the lipid and glucose metabolism. However, coffee intake showed neither association to a lower risk of progression nor in development of DR in clinical studies [44] whereas in preclinical studies showed a protective role inhibiting apoptosis induced by hyperglycemia in retinal tissues [45].

Nutrients in diet can play a massive role in diabetic patients who are resistant to conventional treatment, as these nutritional strategies can reduce the risk of developing DR and attenuate its progression, preserving the normal function and structure of the retina [46]. All these compounds or nutraceuticals have a specific beneficial effect, and when talking about DR, we aim for nutraceuticals that exert their effect on any pathway in the physiopathology of DR [47] as adjuvant therapies.

The DASH diet is based on respecting the following dietary recommendations: limiting sugar-sweetened beverages and sweets; limiting foods that are high in saturated fats, full-dairy products, or tropical oils; consuming vegetables, fruits, whole grains, fat-free or low-fat dairy products, fish, poultry, beans, nuts, and vegetable oils; and thus a greater intake of phytochemicals (see Table 1) [48]. Although this is a dietary pattern related to nutritional management of hypertension, it has shown some beneficial effects on some complications of diabetes. In elderly Korean population, the DASH diet decreases the odds of chronic kidney disease and leads to a lower overall likelihood of diabetic nephropathy (DN), associated with the anti-inflammatory and antioxidant properties of the diet [49] (see Figure 2). In another study, regarding obese population, the DASH diet improved serum antioxidant capacity measured by a higher ferric reducing/antioxidant power of plasma [50]. The DASH diet is a potential strategy for reduction of T2DM and for metabolic control of the disease by improving glycemic and cholesterol control [51, 52]. To our knowledge, there are no studies on the effects of the DASH diet in DR.

The aforementioned diets contain overall healthy food which are rich in some of the specific nutraceuticals we will discuss later. Nonetheless, there is another approach worth mentioning regarding diet and its impact on DR. The low-calorie diet (LCD) is another alternative that has been studied in pro of T2DM (see Table 1). Cohort studies have positively correlated high calorie intake with progression of DR [26]. Calorie restriction slows down age-related deterioration of ocular functions by attenuating mainly OS and improving mitochondrial function [53]. In preclinical studies, a low-carbohydrate diet improved antioxidant biomarkers [54].

The VLCD has been assessed on T2DM patients, with contradictory findings. Reported cases have found this intervention beneficial in glycemic control, but there are no specific trials assessing VLCDs in DR [62, 63]. Nevertheless, caution is needed since VLCDs lower than 600 kcal should only be employed under close clinical supervision [27], and rapid reduction of systemic glucose is associated with worsening of DR [64].

## 4. Supplement Therapies and Diabetic Retinopathy

A wide variety of nutraceuticals, phytochemicals, vitamins, and minerals with antioxidant and/or anti-inflammatory properties, have been shown to play a major role on the onset and progression of DR. Some have been studied as single therapies and others as combined therapies probably because of each one on different points of some, or most, of the routes involved in DR (see Figure 2). Among the pool of options, we considered molecules that have been consistently associated with eye health and/or DR; we also include some relatively new dietary supplements which have shown positive results as promising adjuvant therapies in DR.

### 4.1. Xanthophylls

Xanthophylls are carotenoids that contain oxygen; they are derived from plants as natural pigments and are widely known for their antioxidant properties. Two of the major xanthophylls, containing two hydroxyl groups in their structure and having no effect as provitamin A, are lutein and zeaxanthin. Both substances are found in the fovea; lutein concentration is superior to zeaxanthin which differs from lutein in its double link in one of its hydroxyl groups [1]. These xanthophylls exert a wide variety of effects involved in the genesis of DR. As antioxidants, they act by alternating their single and double links reducing blue light wavelength and protecting the eye from light-induced OS, and in this way, around 90% of blue light is absorbed [65]. Xanthophylls act on glucose and lipid metabolism by the upregulation of peroxisome proliferator-activated receptors (PPARs) and glucose transporters and its effects in the expression of enzymes involved in fatty acid synthesis and cholesterol metabolism [66].

Lutein acts on inflammation by quenching free radicals leading to the blockade of nuclear factor kappa-*β* (NF-*κβ*) pathway activation and by the inhibition of arachidonic acid release, and, in consequence, keeping prostaglandins, thromboxanes, and leukotrienes from being formed [67]. Lutein also can inhibit IL-8 secretion [68] and phosphoinositide 3-kinase (PI3K) activity when it is secondarily increased due to OS [69]. Zeaxanthin was related to restoring vascular endothelial growth factor (VEGF) concentrations in a preclinical study alongside restoring as intercellular adhesion molecule-1 (ICAM-1) comparable to the control group [70].

Astaxanthin is a ketone carotenoid that can bind both from the inside and outside of the cell membrane, a quality that makes it the strongest antioxidant of this family [71]. It can be found in seafood and microalgae (*Haematococcus pluvialis*) [72]. Astaxanthin has proven to be efficient against OS as an antioxidant, in addition to having anti-inflammatory and antiapoptotic properties, underlining its importance in neurodegeneration in DR [73]. In preclinical studies, this xanthophyll has shown to decrease OS damage-induced biomarkers and to increase glutamine synthetase concentrations in Müller cells, reducing apoptosis in retinal ganglion cells as seen in Figure 2 [74, 75]. Astaxanthin can also regulate glycemic states and reduce insulin resistance and exert an anti-inflammatory effect by decreasing the expression of NF-*κβ* and TNF-*α* [76] and by inhibiting the expression of proinflammatory molecules such as ICAM-1 and monocyte chemoattractant protein-1 (MCP-1) and angiogenesis via VEGF [66].

Xanthophylls and other carotenoids have proven to be useful in DR's pathological mechanisms, and they have even shown the ability to diminish OS in retinal tissues when conditioned to chronic hyperglycemic state [68–71, 76–78].

### 4.2. Vitamin C

A water-soluble vitamin that can be found in many natural sources that have been used as capsules and powder in many formulations as a supplement, vitamin C exists in two main forms, ascorbic and dehydroascorbic acid, and it is an important nutraceutical in the aid of many OS-oriented diseases [1]. Ascorbic acid has even been studied in clinical trials regarding obesity in DM alongside pomegranate leaf extract like a bioactive compound [79] and in the improvement of the lipid profile [80]. The main biological functions of ascorbic acid revolve between its ability to act in redox state and as a cofactor for many human enzymes [81, 82]. Ascorbic acid has been found to be in higher concentration in healthy patients, contrary to those with DR who have lower concentrations than patients with DM who have not developed this complication [83] and especially in those who develop diabetic macular ischemia who showed dramatically decreased concentrations compared to nondiabetic controls [84]. This effect is due to its ability to prevent propagation of free radical-induced chain reactions [85], thereby directly scavenging ROS (see Figure 2), preventing breakdown of nitric oxide (NO) and decreasing low-density lipid oxidation [46, 86, 87]. Ascorbic acid also plays a role in angiogenesis (Table 2) by inhibition of VEGF, protecting the endothelial barrier permeability [88].

Ascorbic acid has already been discussed as an adjuvant therapy in many inflammation-mediated disorders because of its anti-inflammatory, immunostimulant, and even antibacterial properties at different doses alongside other medications [89–91], extending its important role to be used as combined therapy rather than monotherapy. Nevertheless, further investigations are needed to assess its efficacy in DR as an adjuvant therapy. Regarding OS, in a clinical study with 1000 mg/day of ascorbic acid, it reduces the activity of the enzyme aldose reductase and thus acts by inhibiting the polyol pathway [92] as shown in Figure 2. Ascorbic acid also prevents the apoptosis of vascular pericytes [93]. It may also have a role in autophagy and apoptosis by induction of autophagosome formation [94], increasing the rate of protein degradation lysosomes [95] and expression Bcl-2 family proteins between hypoxia and reoxygenation status [96].

### 4.3. Vitamin E

Vitamin E is the most important lipid-soluble chain-breaking antioxidant in tissue, red cells, and plasma. The predominant isomer found in the human body is *α*-tocopherol [1, 97]. Lipid metabolism is one of this vitamin's main targets, specifically lipid peroxidation. Vitamin E inhibits malondialdehyde (MDA) formation [98, 99] inducing singlet oxygen, lipid peroxide products, and superoxide radical to form tocopherol radical [100].

Vitamin E can inhibit AGE formation *in vitro*[98], probably due to the protective role that vitamin E exerts on lysosomes to reduce autophagic stress [101]; however, this has only been studied in DN to our knowledge and has yet to show the same properties in retinal tissues.

The dietary intake of vitamin E was associated by Arablou and colleagues with higher catalase enzyme activity [102]. Independently to its antioxidant properties, the *α*-tocopherol, although dose-dependent ranging from 10 to 50 *μ*M, has been shown to be able to inhibit protein kinase C activity [103]. In preclinical studies, *α*-tocopherol increased glutathione reductase (GR) activity, reduced glutathione peroxidase (GPx), and ameliorated total antioxidant capacity [95] and inflammatory response measured with the inflammatory cytokines IL-1*β*, IL-6, and TNF-*α* [104]. Vitamin E can decrease the total diacylglycerol level and thus prevent the abnormal retinal flow like expressed in Figure 2 [105]. Tocotrienol, *in vivo*, also acts as an antiangiogenic agent by decreasing apoptosis of endothelial cells via the growth factor-dependent phosphatidylinositol 3-kinase/PDK/Akt signaling pathway [106]. Even though vitamin E as monotherapy has not proven efficacy on DR in clinical studies [16], in combination with other agents, it may aid DR treatment when targeting both OS and inflammation, though further investigation is still needed.

### 4.4. Zinc

Zinc is the second most abundant trace element in the human body, essential for the structure and function of numerous macromolecules such as lipids, nucleic acids, and enzymes that regulate homeostasis, immune responses, OS, apoptosis, and aging [107, 108]. Zinc can be found in a wide variety of food and beverages in low quantities [109]. A low dietary zinc intake has been associated with a greater likelihood of subretinal fluid in patients with neovascular age-related macular degeneration [110]. Serum zinc levels are significantly lower in patients with DR and even correlate with the severity of DR [111] along with being an independent risk factor for DN [112], thus suggesting its negative effects on the microvasculature.

Zinc acts as a cofactor of the cytosolic and extracellular Zn/Cu superoxide dismutase (SOD) enzyme, which scavenges ROS by catalyzing the dissociation of the O_2_^−^ radical in the less harmful forms O_2_ and H_2_O_2_ [1, 107]. This mineral has a unique role in the phototransduction process and photoreceptor-retinal pigment epithelial interaction, and it induces the expression of metallothioneins which in the long term will function as an effective antioxidant and anti-inflammatory [108].

In preclinical studies, zinc supplementation has shown beneficent effects over OS microvascular damage by clearing free radicals, inhibiting lipid peroxidation, activating metallothionein, and reducing the expression of VEGF [112]. Zinc has a positive relationship with vitamin A levels for it is an essential element in retinol-binding protein [111] and it may also improve the absorption of vitamin E in food [113].

Zinc is needed for its catalytic function in over 100 specific enzymes, indicating the critical role of this element in cellular processes [114], including events of genomic stability, cognitive functions, depression, and OS [115]. Zinc by itself is not actively redox, and therefore, Zn^2+^ does not interact directly with ROS or with free radicals centered on carbon as seen in Figure 2 [116, 117]. Zinc, then, contributes to antioxidant status through its ability to compete with transition metals and copper for binding sites in the cell membrane (Table 1) [118]. Iron and copper ions catalyze the production of lipoperoxides; therefore, their replacement by zinc under conditions of insulin resistance in the plasma membrane could inhibit lipoperoxide formation [119].

Multiple preclinical and clinical studies regarding zinc and DR have been developed with various assessed outcomes such as angiogenesis, where a 3-month therapy with zinc did not alter serum VEGF, brain-derived neurotrophic factor, and nerve growth factor in animal models [120]. In a model of induced insulin resistance in rats, zinc supplementation increased insulin sensitivity and antioxidant capacity, where the antioxidant enzymes catalase, glutathione, GPx, and SOD were diminished in comparison with control models. Zinc supplementation in these animals restored the activity of the enzyme and glutathione synthesis [114]. It also attenuates the OS induced by diabetes in the bloodstream and protects the retina from diabetes-induced increased lipid peroxidation [118, 121]. Concerning inflammation, zinc has multiple mechanisms to ameliorate inflammatory processes such as reducing cytokine production by upregulating the zinc-finger protein A20 and inhibiting NF-*κβ* activation, and consecutively by inhibiting NADPH oxidase, it also prevents the formation of ROS [122]. Blood glucose level is also reduced in T2DM animal models given zinc oxide nanoparticles, with the improvement of glucose metabolism and insulin resistance, in addition to the significant reduction of circulating triglycerides and free fatty acids [123]. Zinc supplementation has a potential ability to hinder diabetes-induced cataracts through downregulation of the polyol pathway [124]; however, it has yet to show a similar effect regarding retinal tissue. Some studies have shown an insufficient dietary consumption zinc, particularly among the elderly [110] that may be associated with increased DNA damage in this population [115].

### 4.5. Manganese

Manganese (Mn) is an essential element present in nature as the fifth most abundant metal in the environment and an essential micronutrient for humans [1] that may be associated with microvascular complications in DM. Mn can be found in a normal diet mostly in legumes, rice, nuts, and whole grains [125]. Only about 5% of the Mn in the diet seems to be absorbed [126]. Mn participates in various mechanisms involved in the genesis of DR, like the generation of ROS by having prooxidative properties and antioxidant mechanisms [127], and it could have a role in inflammation. In fact, the dietary intake of this micronutrient was inversely associated with the incidence of T2DM and was partially associated with lower OS measured by 8-hydroxydeoxyguanosine [128]; nevertheless, in preclinical studies, both deficient or excessive intake of Mn aggravated apoptosis by upregulating capase-3, caspase-8, and caspase-9 and inhibited Nrf2 signaling, while optimal intake protected against ROS, MDA, and protein carbonyl [129]. Mn may have a protective role against endothelial dysfunction by upregulating disulfide bond A-like protein (DsbA-L) and thus increasing adiponectin, which ultimately downregulate ICAM-1, a biomarker of endothelial dysfunction; nonetheless, its evaluation in retinal tissues is necessary [130]. Toxicity is caused mainly because of an excessive dose, primarily from supplementation or nondietary intake; and it has been identified through behavioral abnormalities such as hyperactivity, inferior intellectual function, impaired motor skills, and reduced olfactory function in children [125] and in association with the increase of inflammatory cytokines IL-1*β*, IL-6, and IL-8 and higher methylation of NF-*κβ* member activator NKAP [131].

With regard to OS, manganese superoxide dismutase (MnSOD) is an important mitochondrial antioxidant enzyme in which Mn acts as a cofactor [127]. The Mn is an activator of gluconeogenic enzymes like pyruvate carboxylase and isocitrate dehydrogenase, which protect the mitochondrial membrane through the protective role of SOD in lipoperoxidation, emphasizing its importance in mitochondrial metabolism [132, 133]. In preclinical studies with rats on high-fat diets, Mn improved synthesis and secretion of insulin, which is consistent with the improved mitochondrial function [134]. Also, Mn has various beneficial effects in the aid of different biomechanisms involving different pathologies and specifically in DR, but its usage is still limited due to limited information available in such a specific tissue like the retina.

### 4.6. Alpha-Lipoic Acid

Alpha-lipoic acid is a natural compound found primarily in vegetables and meats (and nowadays in many supplementary components), and it is essential for mitochondrial function, showing a promising role in DR. Also known as thioctic acid, this nutraceutical possesses both hydrophilic and hydrophobic properties, widely distributed through cytosol and cellular membranes [135]. Alpha-lipoic acid is a potent antioxidant, improving insulin sensitivity and fatty acid oxidation by activating AMP-activated protein kinase in diabetic patients [136]. It can inhibit lipid and protein oxidation and ROS scavengers [137]. Lipoic acid induces Nrf2 binding to antioxidant response elements and thus higher gamma glutamylcysteine ligase and its catalytic subunit, and so it ameliorates antioxidative processes related with age [137]. Dihydrolipoic acid can regenerate endogenous antioxidants, such as ascorbic acid, tocopherol, and glutathione [138, 139]. Regarding angiogenesis, in preclinical studies, monotherapy with alpha-lipoic acid decreased the expression of VEGF in cardiac tissue [140], angiopoietin 2, and erythropoietin, protecting the diabetic rat's retina. This is achieved by blocking superoxide formation [141] with the protection of the thickness of ganglion cells [142] and by a probable inhibition of NF-*κβ* (having an important role in inflammation as seen in Figure 2). Alpha-lipoic acid may inhibit endothelial cell apoptosis by activating protein kinase B and upregulating p27 activity [143] (Table 2). Alpha-lipoic acid has many formulations and different administration routes to be assessed on microvascular complications; as an example, an aqueous formulation showed to diminish fluorescein leakage in the eye of streptozotocin-induced diabetic rats [144]. Alpha-lipoic acid's beneficial properties were also assessed in mitochondrial metabolism, as said before, given its importance in DR. Preclinical studies assess mitochondrial function and regulation measured by its transcriptional factor, peroxisome proliferator-activated receptor-*γ* (PPAR-*γ*) coactivator-1*α*, and nuclear respiratory factor 1 (NRF1); a beneficial effect of alpha-lipoic acid was preventing the loss of mitochondrial copy number and increasing gene transcripts of PPAR-*γ* and NRF1 [145]. Clinical and preclinical studies have showed efficacy of lipoic acid as monotherapy or combined antioxidant therapy in DR by measuring different outcomes such as mitochondrial damage through the production of ROS or retinal damage [146, 147]. Clinically, alpha-lipoic acid may have a protective role [148] but this needs to show efficacy on patients who have already developed later stages of DR because it has not shown any effect on macular edema [149].

### 4.7. Curcumin

Curcumin is a unique polyphenol found in turmeric (*Curcuma longa*) and used as spice, food coloring, and traditional medicine due to its antioxidant and anti-inflammatory properties [150]. Curcumin is a crystalline orange-yellow color compound [151]. The World Health Association stated an acceptable daily intake of 0-3 mg/kg as a food additive [152]; yet, unfortunately, turmeric has a poor bioavailability due to poor absorption and rapid metabolism and elimination: even though multiple agents have been used to increase its bioavailability, curcumin has proved to be safe in humans with low toxicity and be well tolerated with doses as high as 12 g/day in clinical trials [153]. This “golden spice” has been thoroughly studied due to its many properties and potential therapeutically targets, being described as pharmacodynamically “fierce” yet pharmacokinetically “weak” by not having conclusive beneficial clinical effects [154].

In diabetes, curcumin has shown to have the ability to increase insulin sensitivity and to exert a hypocholesterolemic and hypoglycemic effect by normalization of triglyceride, cholesterol, OS status, lipid peroxidation, TNF-*α*, and free fatty acids in preclinical studies [155]. In DR, curcumin adjuvant therapy restores retinal antioxidant capacity, increasing antioxidant enzymes and their expression, like SOD and catalase, as well as reducing free radicals [156]. *In vitro*, curcumin showed a potentially synergic effect by boosting MnSOD expression and activity when studied in endothelial progenitor cells [157]. Also, regarding inflammation, curcumin abolishes the expression of important proinflammatory cytokines such as TNF-*α*, VEGF, and ICAM-1 in animal models, and it can inhibit protein kinase C-*β* [155, 158] and ameliorate the inflammatory effects of high-glucose exposure by decreasing proinflammatory cytokines via the Akt/mTOR pathway [159]. These anti-inflammatory properties link curcumin as a potent antiangiogenic agent decreasing VEGF expression (see Figure 2) and playing an important role in DR by inhibiting migration of retinal human endothelial cells by decreasing stromal derived factor-1 [160].

The Nrf2 interacts with Kelch-like ECH-associated protein (Keap-1), the molecule associated with OS-induced damage; however, in response to OS, Nrf2 translocates to the nucleus from cytosol and binds antioxidant/electrophile response element in the promoters of target genes such as NADPH [161]. Curcumin is a promising alternative for the adjuvant therapy in DR although it has yet to show its effects *in vivo* in clinical studies [162, 163].

### 4.8. Polyphenols: Anthocyanins and Resveratrol

A subgroup of flavonoid polyphenols, anthocyanins are water-soluble pigments that give the blue, purple, and red coloration of many fruits and flowers. Among over 20 anthocyanidins that are known, only 6 of them are widely distributed in human diet: cyanidin, delphinidin, pelargonidin, peonidin, malvidin, and petunidin. They can be found primarily in berry fruits from either *Vitaceae* or *Rosaceae* family or dark-colored vegetables and cereals [164]. Anthocyanin metabolism consists mainly in degradation to phenolic acid and subsequently to other stable water compounds [165]. Although anthocyanins were reported to have low bioavailability, their metabolites have been detected in much higher concentrations (by 42-fold) in plasma [166] and have been found in eye-related tissues [167]. These compounds have been studied in different pathologies involving OS and even cancer due to several signaling pathways including mitogen-activated protein kinase, NF-*κβ*, AMP-activated protein kinase, apoptosis, and autophagy [168]. Some of the effects of anthocyanins on DM are as follows: the ability to regulate the expression and translocation of GLUT4 receptor, to increase the activation of PPAR-*γ* and AMP-activated protein kinase, and to inhibit intestinal enzyme *α*-glucosidase and pancreatic *α*-amylase [169]. Anthocyanins have been assessed in preclinical studies exceeding their role as antioxidants in retinal pigment epithelium by neutralizing ROS, inhibiting diabetes-induced retinal abnormalities, and having a protective role in neurodegeneration regarding the retina in *N*-methyl-N-nitrosourea-induced damaged rats [170]. Malvidin and its glycosides increase SOD and catalase in high glucose-induced human retinal capillary endothelial cells, protecting these cells from OS-induced damage, and by anti-inflammatory properties due to inhibition of ICAM-1 and NF-*κβ* (see Table 2) [171]. Cyanidin and orthodihydroxy groups of anthocyanins can inhibit lipid peroxidation by chelating metal ions [172], and other anthocyanins can facilitate nuclear translocation of Nrf2, inducing the activation of this redox-sensitive activation factor [173].

Resveratrol (3,5,4′-trihydroxy-*trans*-stilbene) is another potential adjuvant agent in the aid of DR: resveratrol is an important nonflavonoid polyphenol, the most abundant polyphenol in red wine in concentrations of 8–25 *μ*M, which is a typical component of the Mediterranean diet [42]. Resveratrol has been extensively studied for its wide variety of biological actions such as antioxidative and anti-inflammatory properties important for DR, exerting potentially cardioprotective, neuroprotective, and chemotherapeutic properties [174]. Focusing mainly on DR, resveratrol has a protective role involving OS-induced apoptosis by reducing intracellular ROS through the AMPK/Sirt1/PGC-1*α* pathway [175] and, like the other polyphenols, is as an activator of Nrf2 [173]. In *in vitro* studies with human retinal pigment epithelial cells (ARPE-19 cells), resveratrol showed an inhibitory effect on inflammatory cytokines such as IL-6 and IL-8 [176], and in preclinical studies, resveratrol also inhibited VEGF through yet another pathway, by increasing the activity of paraoxonase 1 (PON1) which is involved in protecting endothelial cells [177] and inhibiting the VEGF expression [178]. Unlike other nutraceuticals, resveratrol exhibits a biological property to act on a more directed manner against apoptosis, mainly on Müller cells by upregulating the microRNA-29b, and so downregulating specific protein 1 expression [179] and directly inhibiting caspase-8 and caspase-3 as seen in Figure 2 [180]. Given all these beneficial effects, more clinical interventions are needed to prove or assess these effects clinically in DR [181].

### 4.9. Ubiquinone

Ubiquinone, also known as coenzyme-Q (CoQ10) is a mobile component of mitochondrial electron transport chain. The CoQ10 is present in all cells and membranes and is necessary for mitochondrial energy production [182]. The richest dietary sources of CoQ10 are meat, migratory fish, and some oils and nuts, and the recommended dose in supplements go from 50 to 150 mg [183]. The CoQ10 is the only lipid-soluble antioxidant that animal cells synthesize *de novo* in the body [184]; and, remarkably, as a combined adjuvant therapy, CoQ10 can recycle and regenerate other antioxidants such as tocopherol and ascorbate [185]. CoQ10 also protects against endothelial dysfunction by activating endothelial nitric oxide synthase and mitochondrial oxidative phosphorylation [185].

As a coadjuvant therapy, it has been assessed alongside the Mediterranean diet in elderly population, and when 200 mg of CoQ10 per day was added, the oxidative and inflammatory states were diminished compared to the Mediterranean and Western diets [186]. A liquid formulation with ubiquinol 100 mg/day was given over 12 weeks in T2DM patients showing increased levels of antioxidant biomarkers like SOD, catalase, and GPx, but not MDA, compared to placebo [187], compared to a similar study done in patients with T2DM and DN where MDA and AGEs levels were diminished [188]. Limited studies have been done in patients with DR regarding the addition of CoQ10. In a clinical study, CoQ10 supplementation for 6 months improved OS state by increasing total antioxidant capacity and decreasing lipid peroxidation [189]. Overall, CoQ10 seems to have a promising therapeutic potential in the aid of diabetes-related complications primarily for its antioxidant properties; and it has been shown to improve visual acuity in patients with age-related macular degeneration, as well as protecting ganglion cell death in glaucoma models [190].

### 4.10. Omega-3 Fatty Acids

Omega-3 is an important product from the Mediterranean diet [33], as it is one of the main reasons why this diet is so commonly healthy. Omega fatty acids encompass three major subtypes: omega-3, omega-6, and omega-9 fatty acids. Among these, omega-3 is subcomposed of three more types: alpha-linoleic acid, eicosapentaenoic acid, and docosahexaenoic acid [191]. Omega-3 fatty acids can be found in fish, and based on observational data, an intake of 500 mg/d is associated with a decreased risk of a sight-threatening DR [33]. This can be explained by several mechanisms. Low-chain PUFAs have an important role for the function and survival of photoreceptors, and they also inhibit cytokine-induced NF-*κβ* activation and translocation, as illustrated in Figure 2. In preclinical studies, they also have shown to decrease inflammation in the eye and their lipoxins, resolvins, and protectins which are derived from these fatty acids that have antiangiogenic capabilities [192, 193]. Preclinical studies have demonstrated that a flaxseed oil diet, another natural source of omega-3 fatty acid, does increase the expression of G-protein-coupled receptor 120 (GPR120), a receptor of omega-3, but not GPR40 [194] (see Table 2). Remarkably, omega-3 PUFAs suppress ER stress in adipocytes by AMPK activation, and resolvin D1, a family derived from eicosapentaenoic and docosahexaenoic acids, attenuates ER stress-induced apoptosis and decreases caspase-3 activity [195].

Overall, omega-3 fatty acids show a remarkable potential in the prevention of progression in DR, primarily in the early stages [196].

### 4.11. Erianin

Erianin (2-methoxy-5-[2-(3c4c5-trimethoxy-phenyl)ethyl]-phenol), a relatively newer nutraceutical not yet explored deeply in DR, is another natural product, derived from *Dendrobium chrysotoxum*, an herb of the Orchidaceae family. Used mainly in Chinese traditional medicine as antipyretic or analgesic, indexed in the Chinese pharmacopoeia, it contains two phenyl rings linked by a 2-carbon bridge with methoxyl substitutions on the phenyl rings [197, 198].

Erianin has been studied mainly for its antiangiogenic properties. In preclinical studies, 100 mg/kg per day of erianin induced growth delay in tumors [199]. In 2015, erianin was first introduced as a DR therapy option, by inhibiting the NF-*κβ* signaling pathway, thus inhibiting retinal inflammation in preclinical studies [198, 199]. Nevertheless, clinical studies are still needed to assess its clinical relevance in DR and its potential use as a concomitant therapy in this disease.

### 4.12. Melatonin

Melatonin, N-acetyl-5-methoxytryptamine, is a ubiquitous molecule found in almost all living organisms. In mammals, melatonin is centrally produced by the pineal gland and is directly released in the blood as a hormone [200], predominantly at night, known to promote sleep products and regulate circadian rhythms. Melatonin has been reported to regulate important molecular pathways related to DR such as apoptotic, antiangiogenic, anti-inflammatory, and Nrf2 pathways by being one of the most powerful antioxidants [200, 201]. The effects of melatonin in reduction of OS are through free radical scavenging activity and stimulating antioxidant enzymes like GR, GPx, SOD, and catalase and by suppressing activity of prooxidant enzymes like nitric oxide synthase and its inducible form (iNOS) and cyclooxygenase-2 [202]. In preclinical studies with streptozotocin-induced prediabetic rats, 85 *μ*g/animal/day of melatonin improved fasting glucose and serum insulin levels and had a significant decrease in retinal thiobarbituric acid-reactive substances, iNOS, VEGF, and matrix metalloproteinase 9 [203]. In another study in a model with oxidative glutamate toxicity in combination with buthionine sulfoximine, intravitreal injections of melatonin showed a protective effect against apoptosis of retinal ganglion cells, acting as a neuroprotective agent [204] (see Figure 2). Lower salivary melatonin peak levels have been reported in patients with DM compared to nondiabetic patients [205]. Melatonin can ameliorate inflammation, reducing inflammatory cytokines and iNOS through inhibition of NF-*κβ*, and may reduce ER-induced apoptosis and autophagy [202].

As an adjuvant combined therapy alongside zinc acetate, it has shown to improve postprandial and fasting glucose levels [206]. More clinical interventions are needed to assess its effect in DR: melatonin shows a promising beneficial effect in this disease, as summarized in Table 2.

### 4.13. Combined Therapies

**Figure 3:**
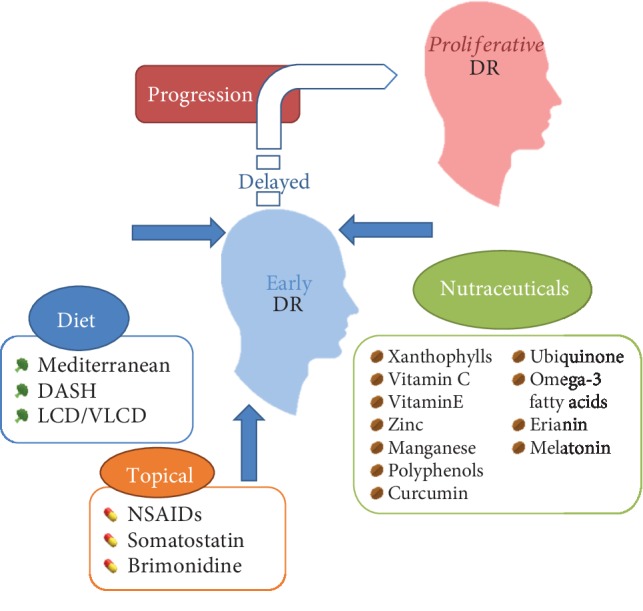
A model of nontraditional therapies focused for diabetic retinopathy to diminish its progression from early to proliferative stages. Represents the two main topics focusing on diet and nutraceuticals either alone or combined, both as an alternative adjuvant therapy for DR. Abbreviations: DR: diabetic retinopathy; DASH: Dietary Approaches to Stop Hypertension; LCD: low-calorie diet; VLCD: very low-calorie diet; NSAIDs: nonsteroidal anti-inflammatory drugs.

The approach of combined therapies (see Figure 3) as an adjuvant therapy for early stages of DR is to combine the best possible nutraceuticals aimed at the key mentioned biomechanisms such as OS and inflammation, to delay progression to PDR without leaving behind the established glycemic control. Many options have been already rehearsed in clinical stages (shown in Table 3), even using compounds not mentioned in this review, like *Ginkgo biloba*, which may have a protective role in early DR [4, 207]. Nevertheless, preclinical approaches assessing different combinations have not shown statistical significance on their primary outcomes in clinical studies whereas some preclinical studies do. When considering new therapeutic strategies to treat DR, targeting a different mechanism other than VEGF should be the aim [178] to focus on, given just one single option may not be the most optimal for our patients. A recent meta-analysis regarding the use of Chinese herbs as a single adjuvant therapy for DR, like erianin, does not conclude that a single therapy can be beneficial by itself. However, a combined use of drugs may improve the synergic effect, supporting the use of combined adjuvant therapies as a field to be explored for the management of DR [4, 208]. A mixture of supplements, reviewed by Tabatabaei et al., may have an additive effect, diminishing mitochondrial dysfunction, OS, and endothelial damage [209]. More clinical interventions are needed to assess possible therapeutic combinations for DR treatment considering modifications in OS, inflammation, neovascularization, neurodegeneration, and mitochondrial damage pathways.

### 4.14. Nonsteroidal Anti-Inflammatory Drugs

Inflammation has proven to be an essential mechanism in the genesis of DR. In a recent study in vitreous tissue of patients with DR, inflammation biomarkers such as IL-1*β*, TNF-*α*, IL-18, IL-6, caspase-1, interferon gamma (IFN-*γ*), prostaglandin E_2_ (PGE_2_), and VEGF were increased in comparison to nondiabetic patients [223]. This emphasizes the therapeutic potential of nonsteroidal anti-inflammatory drugs (NSAIDs) as an adjuvant therapy in DR.

Topical NSAIDs are delivered through the posterior segment from 2 different routes: corneal and noncorneal pathways. The first one is limited due to precorneal space by lacrimation and ionization of acids in the pharmaceutical compounds, and also, the reduced fraction that reaches the anterior chamber is cleared by the physiologic flow of aqueous humor, and finally, the iridolenticular diaphragm creates a concentration gradient limiting even more its penetration to the posterior chamber. The noncorneal pathway involved penetration through the conjunctiva and sclera, which may be a possible route to uveal and to retinal tissues [224]. Even with this complexity, topical NSAIDs such as indomethacin, bromfenac, and nepafenac have all shown to reduce PGE_2_ levels in prospective clinical studies [225]. Topical nepafenac has been the most evaluated NSAID, to our knowledge. When evaluated in noncentral diabetic macular edema, nepafenac showed no statistical difference in retinal volume measured by optical coherence tomography with a therapy consisting of 3 applications daily for 12 months [226]. Nevertheless, applying 3 times daily in one eye did show a reduction effect in retinal thickness in another shorter study alongside a narrowing effect on the retinal arteriole diameter from the first week of treatment [227]. The use of NSAIDs is safe and well tolerated. Corneal epithelial damage and punctate keratitis are mainly sporadic adverse side effects [228] associated with topical ophthalmic medications containing preservatives; however, adverse side effects could be more common in patients with history of complicated corneal surgery, diabetes, dry eye syndrome, and rheumatoid arthritis [224].

### 4.15. Other Adjuvant Therapies

Similar to NSAIDs, other systemic medications have been assessed in DR. Having the clinical perspective of a T2DM patient whose progression of the disease has reached development of DR, it is expected that these patients are already taking several medications whether for DM itself or related disorders from metabolic syndrome, like dyslipidemia or hypertension. A recent meta-analysis of glucose-lowering medications indicated an increased risk of DR with sulfonylureas, and there is no association with dipeptidyl peptidase 4 inhibitors (DPP-4i), glucagon-like peptide-1 receptor agonist (GLP-1RA), and sodium-glucose transport protein 2 (SGLT2); still, the latter was associated with the lowest probability to develop DR complications [229]. Regarding dyslipidemia, contradictive information has been found with its association to DR and its complication as shown from a meta-analysis that even though lipid profile was worse in patients who developed diabetic macular edema, no higher risk to develop a more severe stage nor worsening of hard exudates was shown [226]. Nonetheless, in recent cohort studies, statin therapy was associated with a decreased risk of DR and its complications compared to a population not taking statins [230, 231].

Another pathological implication involved in the genesis of DR is the renin-angiotensin-aldosterone system (RAAS), more specifically, angiotensin II and aldosterone which are involved with retinal angiogenesis, vascular leakage, neurovascular regulation, edema, and inflammation [232, 233]. This leads to the hypothesis of the usage of RAAS inhibitors in DR as a possible therapy option; and in normotensive patients, either angiotensin-converting enzyme inhibitors or angiotensin receptor blockers showed a decreased risk for DR progression and increased possible regression, but this was not the case with hypertensive patients [234].

Fenofibrates have also been studied in DR in clinical and preclinical studies. In a preclinical study, fenofibrate attenuated OS and inflammation in Müller cells by activating Nrf2 (as seen in Figure 2) and by inhibiting nucleotide-binding domain, leucine-rich repeat-containing receptor, and pyrin domain-containing 3 (NLRP3) inflammasome activation [235]. In a retrospective cohort study, fibrates were associated with a reduced progression of DR [236] and also protection of the retinal nerve fiber layer loss associated with early neurodegeneration in DR, by showing a thicker layer compared to T2DM patients who did not take fibrates [237]. A review regarding the usage of fenofibrates in DR showed a beneficial effect independently to its ability to lower lipids or triglycerides and recommended a maximum dose of 200 mg per day, or 67 mg if impaired kidney function is present, though fenofibrates are contraindicated in severe stages of DN [238]. Fenofibrates may have the potential to reduce the progression of DR, but more information is needed to either confirm or negate their efficacy [239, 240].

An approach that stands out is the administration of topical neuroprotective agents for DR, primarily somatostatin and brimonidine, again highlighting the importance of neurodegeneration in DR. The hypothesis involving the inhibition of growth hormone and insulin-like growth factor 1 (IGF-1) that could diminish DR progression began in the early 2000s but caught more attention after the EUROCONDOR study [241]. Somatostatin analogues may play a role in DR by preventing neovascularization, vascular leakage, and neurodegeneration independently to IGF-1 [242]. Brimonidine, on the other hand, is a drug aimed at lowering intraocular pressure in patients with ocular hypertension by improving microcirculation and increasing ocular perfusion [243]. It also diminishes cell injury through elimination of glutamate-induced excitotoxicity of *N*-methyl-D-aspartate receptors [244]. Both drugs by topical administration showed a protective effect on DR; somatostatin did so by dilating central retinal arteriolar equivalent, and venular dilation with both drugs in EUROCONDOR's 2-year therapy [245], and it may prevent worsening of preexisting neurodysfunction [246].

### 4.16. Future Perspectives

Different adjuvant therapies have been evaluated in this review focusing mainly on diminishing the progression of DR, through other routes, such as using light stimulation or pharmacological strategies to shift photoreceptors into less energy-consuming metabolic states [247]. The complexity to deliver cost-effective drugs to the posterior segment of the eye has led to novel approaches [248]. A new drug delivery system with sustained-release implants, nanotechnology [249, 250], and lysosomes is being developed. A combination therapy of novel inhibitors targeting the molecules beyond VEGF may be more effective in treating DME in the coming years, such as the Kinin-Kallikrein system or Tie-2, the latter being the receptor tyrosine kinase of angiopoietins 1 and 2 [251, 252].

In addition, different technologies such as intravitreal implants, devices in the suprachoroideal space, or use of magnetic field to deliver drugs to the posterior segment of the eye have been reviewed with promising results, which have yet to show efficacy in further studies [248]. Ultimately, the aim is to be able to offer a cost-effective and noninvasive therapy to prevent DR consequential blindness, aiming at neovascularization [253] or as shown in this article inflammation and/or OS (preferably in a more localized manner is the future in the management of DR). An example of this is the encapsulated form in liposomes of anthocyanins which increased their bioefficacy [166] or gold-coated nanoparticles of resveratrol which showed a beneficial effect by lowering inflammatory cytokines and decreasing angiogenesis in streptozotocin-induced diabetic rats [254]; more biomarkers are being studied, such as pentraxin 3, which is another serum biomarker of DR that was associated with its development and progression with high sensitivity levels, indicating more advanced stages of the disease [255], broadening the horizon in the management of DR.

## 5. Conclusion

DR is a progressive disease, a consequence of DM that can ultimately lead to visual disability. The physiopathology of DR is involved in a complex variety of biochemical pathways such as OS and inflammation as main therapeutic targets and also neurodegeneration, apoptosis, autophagy, lipid metabolism abnormalities, ER stress, and ultimately angiogenesis. The approach of this article is to focus on the already-known diets, nutraceuticals, and different compounds as an adjuvant approach in the treatment DR to diminish its progression.

We expect to focus our future perspectives for interventions assessing different combined adjuvant therapies in the pathways described in this multifactorial disease, to simultaneously stop or diminish the progression of DR from early stages to more severe ones preventing the visual impairment in patients with DM.

## Figures and Tables

**Table 1:** Diet compositions and servings in DR. In this table, we exemplify the different diets mentioned above with their compositions and servings. A row with the average American diet was added in order to contrast its composition to the recommended dietary regimens.

Diet	Contents (daily average)	Servings	Outcomes
Dietary Pattern to Stop Hypertension	(i) Flavonols 3.53 ± 2.39 (mg/1000 kcal) (ii) Flavones 0.13 ± 0.06 (iii) Flavonones 6.77 ± 1.37 (iv) Flavan-3-ols 2.93 ± 2.70 (v) Anthocyanidins 0.33 ± 0.00 (vi) Carotenoids 12.24 ± 6.94 (vii) Phytosterols 47.19 ± 22.48 [54]	Daily: (i) 4-5 fruit servings (ii) 4-5 vegetable servings (iii) 7-8 servings of grains or their products (iv) 2-3 low-fat or fat-free dairy servings (v) 2 or less servings of meats, poultry, or fish (vi) 2-3 servings of fats and oils Weekly: (i) 4-5 servings per week of nuts, seeds, or dry beans (ii) 5 servings of sweets Recommended sodium intake is roughly 2400 mg per day [55]	*Clinical outcomes:* ↑ Glycemic and metabolic control in DM patients [27–29, 51, 52] ↓ Incidence of DN [49] *Biomarkers:* ↓ ACR, FBG, HbA1c, TC, and serum Cr [49] ↑ Plasma antioxidant capacity ↔ F_2_-isoprostanes [50].

Mediterranean	(i) Flavonols 184.89 (ii) Flavones 61.00 (iii) Flavonones 269.12 (iv) Anthocyanidins 77.3 [56]	In every meal: (i) 1-2 fruit servings (ii) 2 or more vegetable servings (iii) Olive oil (iv) 1-2 servings of bread/pasta/rice/couscous/other cereals Daily: (i) 1-2 servings of olives/nuts/seeds (ii) Herbs/spices/garlic/onions (less salt) (iii) 2 servings of dairy products (preferably low fat) Weekly: (i) 2 servings of white meat (ii) 3 or less servings of potatoes (iii) 2 or less red meat servings (iv) 2-4 eggs (v) 2 or more servings of legumes (vi) 2 or less servings of sweets Wine in moderation [57]	*Clinical outcomes:* Play a protective role in DM [34–37] and its microvascular complications such as DR [31, 32] *Biomarkers:* ↓ ACR, FBG, HbA1c, TC, and serum Cr [49] ↓ MCP-1, NF-*κβ*, IL-8, VCAM, and ICAM ↓ TNF-*α*-induced VEGF expression, ROS-induced lipid peroxidation ↑ Nrf2 protection against OS [39, 42]

Low-calorie diets	May be as strict as 600 kcal Carbohydrates may go from 20 to 60 g/day Consumption of protein and fat is increased to compensate [58]	Example of one-day low-calorie diet Breakfast: coffee (12 oz), cottage cheese (1.5 cup), and fruit cocktail (0.5 cup) Morning snack: medium apple, medium banana Lunch: medium apple, bread whole wheat slice (2), cheddar cheese (2 cubic inches), mayonnaise (0.15 cup), and turkey breast/white meat (3 oz) Afternoon snack: bread slice rye 7 grain (2), any fruit-flavored jelly (4tsp), and peanut butter (2 tbsp) Dinner: chicken breast/white meat (4 oz); rice: white cook steamed (1.5 cups); low-calorie dressing (salad); mayonnaise (4tbsp); croutons plain (0.25 cup); and 1 small garden salad with tomato and onion [59]	*Clinical outcomes:* Decreased risk of progression to DR [26] Independently, carbohydrate intake is not associated with DR or other complications of DM [30] *Biomarkers:* ↑ Glutathione, GPx, GR, and ascorbic acid [53, 54]

Average American diet	(i) Flavonols 2.24 ± 1.47 (ii) Flavones 0.16 ± 0.08 (iii) Flavonones 0.44 ± 0.00 (iv) Flavan-3-ols 1.67 ± 1.12 (v) Anthocyanidins 0.22 ± 0.00 (vi) Carotenoids 3.81 ± 1.73 (vii) Phytosterols 19.22 ± 10.69 [54] (viii) 2200 calories–2070 kcal [60] (ix) 275 carbohydrate g/day [58] (x) 3600 mg/day of sodium [61]		

Abbreviations: DM: diabetes mellitus; DR: diabetic retinopathy; DN: diabetic nephropathy; OS: oxidative stress; ACR: albumin-creatinine ratio; FBG: fasting blood glucose; HbA1c: glycated hemoglobin; TC: total cholesterol; Cr: creatinine; MCP-1: monocyte chemoattractant protein-1; NF-*κβ*: nuclear factor kappa-*β*; IL-8: interleukin 8; VEGF: vascular endothelial growth factor; VCAM: vascular cell adhesion molecule; ICAM: intercellular adhesion molecule; GPx: glutathione peroxidase; GR: glutathione reductase.

**Table 2:** Relationship between nutraceuticals and the pathophysiological pathways of DR. The “✓” on each cell indicates whether the nutraceutical (rows) has shown an effect on each mechanism (columns). In this table, it is remarkable how every nutraceutical has at least some antioxidant effect. Since various mechanisms are involved in DR, we can then aim the therapeutic approach to several targets. ^∗^High doses may be prooxidant instead of antioxidant.

Diabetic retinopathy						
Nutraceutical	Oxidative stress	Inflammation	Angiogenesis	Apoptosis and autophagy	Antidiabetes properties	Endoplasmic reticulum stress
Xanthophylls	✓	✓	✓			
Vitamin C	✓		✓	✓		
Vitamin E	✓		✓		✓	
Zinc	✓	✓				
Alpha-lipoic acid	✓	✓	✓	✓		
Manganese	✓^∗^				✓	
Curcumin	✓	✓	✓		✓	
Polyphenols and resveratrol	✓	✓	✓	✓	✓	
Ubiquinone	✓			✓	✓	
Erianin	✓	✓	✓			
Omega-3 fatty acids	✓	✓	✓			✓
Melatonin	✓	✓	✓	✓		✓

**Table 3:** Clinical and preclinical studies using combined antioxidant therapies. Although they do not share the same primary outcomes, these studies all aim to establish significance in the treatment of the onset of DR, sharing the thought of looking for a nontraditional but effective adjuvant therapy to diminish DR progression, showing purely combined antioxidant therapies and adjuvant to metformin or statins.

Design	Intervention	Results	Reference
Clinical trial	(i) Group 1: zinc (20 mg), magnesium (250 mg), vitamin C (200 mg), and vitamin E (100 mg) (ii) Group 2: zinc, magnesium, vitamin C, vitamin E, vitamin B_1_ (10 mg), vitamin B_2_ (10 mg), vitamin B_6_ (10 mg), biotin (200 *μ*g), vitamin B_12_ (10 *μ*g), and folic acid (1 mg) (iii) Group 3: placebo	No difference was observed between the three groups. Short-termed combined adjuvant therapy did not show efficacy in MNSI score for diabetic neuropathy	[210]

Clinical trial	(i) Group 1: oral glucose (75 g) (ii) Group 2: oral glucose, vitamin C (2 g), and vitamin E (800 IU)	FMD did not change significantly after glucose plus vitamins.	[211]

Clinical trial	12 weeks of 6 d/week of exercise with: (i) Group 1: vitamin E (400 IU of D-*α*-tocopherol) plus 3 tea sachets 3 per/d in 240 mL of water containing 1.5 g per sachet (ii) Group 2: placebo	There was a decrease of waist circumferences and fasting glucose level; erythrocyte catalase activities increased in the combined adjuvant therapy group.	[212]

Clinical trial	(i) Group 1: vitamin E (800 IU/d), vitamin C (500 mg/d), and *α*-lipoic acid (900 mg/d) (ii) Group 2: CoQ10 3 (400 mg) 3 per day (iii) Group 3: placebo	Antioxidants did not alter the cerebrospinal fluid biomarkers	[213]

Clinical trial	(i) Group 1: Ginkgo biloba dry extract (ii) Group 2: *α*-lipoic acid and vitamin C (iii) Group 3: papaverine chlorhydrate and vitamin E (iv) Group 4: placebo	There was no statistical difference in audiometry, speech recognition threshold, or percentage index of speech recognition in patients with presbycusis	[214]

Clinical trial	(i) Group 1: CoQ10 (400 mg) (ii) Group 2: lutein (10 mg), astaxanthin (4 mg), zeaxanthin (1 mg), vitamin C (180 mg), vitamin E (30 mg), zinc (20 mg), and copper (1 mg). (iii) Group 3: placebo	Mitochondrial dysfunction measured by SMF of platelets and its hydrolytic activity increased in the CoQ10 and combined therapy group	[215]

Clinical trial	(i) Group 1: intravitreal ranibizumab (0.5 mg/0.05 mL) (ii) Group 2: intravitreal ranibizumab with the added DHA supplementation group which is composed of the following: concentrated oil in omega-3 fatty acids (500 mg), triglyceride-bound (350 mg), eicosapentaenoic acid (42.5 mg), docosapentaenoic acid (30 mg), vitamin B_1_ (0.37 mg), vitamin B_2_ (0.47 mg), vitamin B_3_ (5.3 mg NE), vitamin B_6_ (0.47 mg) vitamin B_9_ (66.7 *μ*g), vitamin B_12_ (0.83 *μ*g), vitamin C 26.7 mg), vitamin E (4 mg), zinc (1.66 mg), copper (0.16 mg), selenium (9.16 *μ*g), manganese (0.33 mg), lutein (3 mg), zeaxanthin (0.3 mg), and glutathione (2 mg)	Statistically significant decrease of central subfield macular thickness in favor of the combined DHA supplementation group, but improvement in the best-corrected visual acuity measured in ETDRS was not statistically significant.	[216]

Preclinical	(i) Group 1: normal diet with added supplementation of the following: vitamin C (300 mg), vitamin D_3_ (10,000 IU), vitamin E (300 IU), fish oil (1.6 g), eicosapentaenoic acid (650 mg), docosahexaenoic acid (500 mg), benfotiamine (1 g), *α*-lipoic acid (750 mg), tocomin (200 mg), zeaxanthin (40 mg), lutein (20 mg), resveratrol, green tea, turmeric root (curcumoids), N-acetyl-cysteine, Pycnogenol® pine bark, grape seed extract, CoQ10, zinc (2.65 g), and soybean oil (ii) Group 2: no supplementation	The group receiving the adjuvant supplement showed decreased capillary cell apoptosis, attenuated retinal damage, OS, mitochondrial damage, and inflammation with no impact on hyperglycemia	[217]

Preclinical	(i) Group 1: control group (ii) Group 2: diabetic rats left for 3 days (iii) Group 3: untreated diabetic rat group (iv) Group 4: diabetic rats treated with CoQ10 (10 mg/kg b.wt.) (v) Group 5: diabetic rats treated with niacin (40 mg/kg b.wt.) (vi) Group 6: CoQ10 and niacin (vii) Group 7: glibenclamide (5 mg/kg b.wt.) (viii) Group 8: donepezil hydrochloride (3 mg/kg b.wt.) (ix) Group 9: glibenclamide and donepezil hydrochloride	CoQ10 and niacin improved glucose and insulin levels, an improvement of neurotransmitters and OS biomarkers. Decrease in levels of ICAM, VCAM, and Ang-II and finally decreased levels of TNF-*α* and caspase-3	[218]

Preclinical	(i) Group 1: control group (ii) Group 2: DM and streptozotocin vehicle (iii) Group 3: DM+MPO (2 mg/kg bw) (iv) Group 4: DM+MPO (10 mg/kg bw) (v) Group 5: DM+MPO (50 mg/kg bw)	MPO groups showed a reduced aldose reductase activity and reduced expression of p38MAPK and ERK1/2 in rat lens. Medium (10 mg/kg bw) dosing showed a significant decrease in GPx activity in lens of diabetic rats	[219]

Preclinical	(i) Group 1: ascorbic acid (100 mg/kg) (ii) Group 2: ascorbic acid (200 mg/kg) (iii) Group 3: diclofenac sodium (5 mg/kg) (iv) Group 4: diclofenac sodium and ascorbic acid (100 mg/kg) (v) Group 5: diclofenac sodium and ascorbic acid (200 mg/kg) (vi) Group 6: prednisolone (5 mg/kg) (vii) Group 7: prednisolone and ascorbic acid (100 mg/kg) (viii) Group 8: prednisolone and ascorbic acid (200 mg/kg) (ix) Group 9: atorvastatin (8 mg/kg) (x) Group 10: atorvastatin and ascorbic acid (100 mg/kg) (xi) Group 11: atorvastatin and ascorbic acid (200 mg/kg) (xii) Group 1: control	The formalin- and carrageenan-induced inflammation was best inhibited by the combination of diclofenac and ascorbic acid	[220]

Preclinical	(i) Group 1: low-fat diet (ii) Group 2: high-fat diet treated with metformin (250 mg/kg/day) (iii) Group 3: high-fat diet treated with resveratrol (100 mg/kg/day) (iv) Group 4: high-fat diet treated with metformin and resveratrol	Significant restoration of AMPK with combined therapy. Metformin alone did not induce AMPK activation in prefrontal cortex	[221]

Retrospective	(i) Group 1: statin consumption (ii) Group 2: statin with vitamin C	Statins decrease complication rate of NPDR, and an increased protective effect when vitamin C was added	[222]

Clinical trial	(i) Group 1: placebo and metformin (2550 mg/day) (ii) Group 2: melatonin (10 mg), zinc (50 mg), and metformin (iii) Group 3: melatonin and zinc	Melatonin and zinc, alone or adjunct to metformin improved fasting and postprandial glucose levels	[206]

Abbreviations: MNSI: Michigan Neuropathy Screening Instrument; FMD: flow-mediated dilatation; SMF: submitochondrial membrane fluidity; ETDRS: Early Treatment Diabetic Retinopathy Study; CoQ10: coenzyme-Q; OS: oxidative stress; ICAM-1: intercellular adhesion molecule-1; VCAM-1: vascular cell adhesion molecule-1; TNF-*α*: tumor necrosis factor-alpha; DM: diabetes mellitus; MPO: *Mangifera indica* L. and *Polygonum odoratum* L. (extract); p38MAPK: p38 mitogen-activated protein kinase; ERK1/2: extracellular signal-related protein kinase 1/2; GPx: glutathione peroxidase; AMPK: AMP protein kinase; NPDR: nonproliferative diabetic retinopathy.
